# Supplementary material for: Lifelong regeneration of cerebellar Purkinje cells after induced cell ablation in zebrafish
Source: eLife. 2023 Apr 12;12:e79672. doi: 10.7554/eLife.79672 (PMC10147380; doi:10.7554/eLife.79672)
Supplement: Supplementary file 1. — Mean, standard deviation, statistic test applied, ‘n’ number, p value, and level of significance are indicated. The reference to the different figures and groups of the comparisons performed in each figure are highlighted in bold. Abbreviations: 4-OHT 4-hydroxy-tamoxifen, BrdU Bromodeoxyuridine, DMSO dimethyl sulfoxide, dpf days post-fertilization, dpt days post-treatment, EdU Ethynyl-2′-deoxyuridine, Endox Endoxifen, EtOH ethanol, gfap glial fibrillary acidic protein, mpf months post-fertilization, mpt months post-treatment, NTT novel tank test, OKR optokinetic response, PC Purkinje cells, PCL Purkinje cell layer, ptf1a pancreas-associated transcription factor 1a, Tg transgenic, wpt weeks post-treatment, WT wild-type, ypf years post-fertilization, ypt years post-treatment. [file elife-79672-supp1.docx]

**Supplementary File 1.**

**Table of detailed statistics, related to Figures 1-7 and respective figure supplements.**

Statistical tests applied, mean standard deviation and n-numbers are indicated, level of significant differences and P values are indicated.

| **FIGURE** | **comparison** | **Mean ± standard deviation** | **Statistics , Post-hoc test** | **p-values , significance** |
| --- | --- | --- | --- | --- |
| **1C (PCs)** | Number PCs (0dpt-11dpt) (n=4-9) | **4-OHT:** 62.17±29.03, 28.83±24.17, 14.5±6.95, 15.2±2.049, 13.75±7.762, 39.2±4.147, 81.33±26.33, 98.5±29.55, 208.5±55.66  **EtOH:**288.3±25.51, 328±56.28, 341.7±40.69, 382.8±39.32, 364.3±50.49, 396.3±56.67, 396.8±8.438, 419.3±38.09, 460.2±38.42 | Unpaired t-test, two tailed | **4-OHT vs EtOH:** <0.0001 (****), <0.0001 (****), <0.0001 (****), <0.0001 (****), <0.0001 (****), <0.0001 (****), <0.0001 (****), <0.0001 (****), <0.0001 (****) |
| **1E (PCs)** | Number PCs (0dpt-6mpt) (n=5-6) | **4-OHT**:77±7.294, 46±11.25,71.4±17.88, 158.3±29.43, 188±13.78, 896.8±94.18, 1248±280.3, 1458±140.9  **EtOH:**351.6±33.44, 367.4±34.77, 394±40.29, 396±53.39, 408.4±67.91, 1277±358.3, 1368±235.6, 1413±83.47 | Unpaired t-test, two tailed | **4-OHT vs EtOH:** <0.0001 (****), <0.0001 (****), <0.0001 (****), <0.0001 (****), 0.0001 (***), 0.0508, 0.4678, 0.5442 |
| **2B (*gfap*)** | number *gfap+* cells (0-8 dpt) (n=4-6) | **4-OHT:**99±22.2, 88,2±24.71, 86±45.58, 79±47.63, 103.4±11.78, 87.6±18.85, 62.2±9.445, 73.75±33.33, 58.17±16.23  **EtOH:**85.8±35.15, 86.8±20.17, 81.8±41.53, 88.8±34.65, 87±10.88, 82±17.12, 69.4±14.64, 71,6±34.89, 77.17±23.03 | Unpaired t-test, two tailed | **4-OHT vs EtOH:** 0.4979, 0.9242, 0.8777, 0.7195, 0.04 (*), 0.6361, 0.3824, 0.928, 0.1295 |
| **2G (%PCs-*ptf1a*)** | %PCs RFP+/GFP+ (0-23 dpt) (n=4-7) | **4-OHT:**1.33±0.542, 1.2±0.351, 3.43±0.506, 3.62±0.379, 4.86±0.452, 5.21±0.658, 7.53±0.561, 7.72±0.376, 11.96±0.717  **EtOH:**8.02±0.897, 9.08±0.887, 8.3±0.842, 8,53±0.775, 7.79±0.87, 7.55±0.372, 8.39±0.952, 8.91±0.732, 10.58±0.721 | Unpaired t-test, two tailed | **4-OHT vs EtOH:** 0.6445, 0.1116, 0.0006 (***), 0.0007 (***), 0.0034 (**), 0.0012 (**), 0.0016 (**), 0.009 (**), <0.0001 (****), <0.0001 (****), 0.0178 (*), 0.4462 |
| **3C (patch clamp)** | Average firing frequency of Purkinje cells (3-21 dpt) (n=18-45) | **4-OHT:** 1.33±0.542 (n=23), 1.20±0.351 (n=35), 3.43±0.506 (n=42), 3.62±0.379 (n=44), 4.86±0.452 (n=41), 5.21±0.658 (n=32), 7.53±0.561 (n=37), 7.72±0.376 (n=39), 11.96±0.717 (n=37)  **EtOH:**8.02±0.897 (n=20), 9.08±0.887 (n=21), 8.3±0.842 (n=23), 8.53±0.775 (n=21), 7.79±0.870 (n=19), 7.55±0.372 (n=18), 8.39±0.952 (n=18), 8.91±0.732 (n=27), 10.58±0.721 (n=28) | 2-Way ANOVA, Šídák's multiple comparisons test | **4-OHT vs EtOH:** <0.0001 (****), <0.0001 (****), <0.0001 (****), <0.0001 (****), 0.0154 (*), 0.1498, 0.9858, 0.7778, 0.6234 |
| **3D (patch clamp)** | Average highest burst frequency of PCs (3-21 dpt) (n=18-44) | **4-OHT:**7.73±2.8 (n=23), 7.14±2.14 (n=35), 16.2±2.21 (n=42), 21.77±2.11 (n=44), 27.19±1.86 (n=43), 22.06±2.36 (n=32), 25.61±2.02 (n=38), 23.63±1.83 (n=43), 27.1±1.42 (n=40)  **EtOH:**31.58±4.26 (n=26), 31.22±3.14 (n=23), 28.12±3.53 (n=25), 28.18±2.98 (n=22), 28.11±2.88 (n=19), 24.73±1.83 (n=22), 23.17±3.33 (18), 20.69±1.68 (n=26), 17.19±0.97 (n=31) | 2-Way ANOVA, Šídák's multiple comparisons test | **4-OHT vs EtOH:** <0.0001 (****), <0.0001 (****), 0.0031 (**), 0.4355, >0.9999, 0.9963, 0.9985, 0.9838, 0.0149 (*) |
| **3E (patch clamp)** | Average cs/ss ratio of PCs (3-21 dpt) (n=17-42) | **4-OHT:** 0.0836±0.0338 (n=23), 0.0906±0.0473 (n=34), 0.1437±0.0331 (n=42), 0.2111±0.0464 (n=42), 0.0972±0.0198 (n=40), 0.0609±0.0169 (n=30), 0.0531±0.0118 (n=36), 0.0429±0.0061 (n=39), 0.0082±0.0017 (n=37)  **EtOH:**0.0399±0.0041 (n=19), 0.0313±0.0064 (n=19), 0.0384±0.0077 (n=18), 0.0381±0.0069 (n=18), 0.0458±0.0114 (n=17), 0.0377±0.0065 (n=16), 0.0436±0.0134 (n=26), 0.0166±0.0036 (n=26), 0.0110±0.0027 (n=26) | 2-Way ANOVA, Šídák's multiple comparisons test | **4-OHT vs EtOH:** 0.9716, 0.7676, 0.0821, 0.0002 (***), 0.8772, 0.9997, >0.9999, 0.9966, >0.9999 |
| **3H (patch clamp)** | Average firing frequency of Eurydendroid cells(014 dpt) (n=27-43) | **4-OHT:**3.716±0.474 (n=18), 3.886±0.397 (n=24), 3.626±0.298 (n=28), 6.473±0.551 (n=34), 6.466±0.786 (n=32), 6.293±0.576 (n=34), 6.628±0.461 (n=40)  **EtOH**: 2.458±0.289 (n=17), 3.491±0.517 (n=22), 2.791±0.398 (n=22) 4.382±0.448 (n=23), 4.832±0.476 (n=24), 6.965±0.725 (n=23) 6.748±0.765 (n=24) | 2-Way ANOVA, Šídák's multiple comparisons test | **4-OHT vs EtOH:** 0.2012, 0.9961, 0.5238, 0.0328 (*), 0.4484, 0.9884, >0.9999 |
| **4D (OKR)** | speed eye rotat (2, 10dpt) (n=27-28) | **4-OHT:** 2.969±1.639, 4.907±1.730;  **EtOH:** 5.236±1.421, 4.721±1.919 | Mann-Whitney test, two tailed (speed eye rotation 2dpt)  Unpaired t-test, two tailed (speed eye rotation 10dpt) | **4-OHT vs EtOH (2dpt):**  <0.0001 (****)  **4-OHT vs EtOH (10dpt):**  0.7074 |
|  | number saccades (2, 10dpt) (n=27-28) | **4-OHT:**3.179±2.310, 5.571±2.284;  **EtOH:**5.893±2.315, 5.22±2.607 | Unpaired t-test, two tailed (number saccades 2, 10dpt) | **4-OHT vs EtOH:** <0.0001 (****), 0.5991 |
| **5C (swimming 12 well plate)** | %time middle zone (2-11dpt) (n=78-135) | **4-OHT-Tg:** 6.581±6.132, 14.54±14.38;  **4-OHT-WT**: 12.13±12.63, 16.45±12.28;  **EtOH-Tg:** 11.26±10.55, 14.6±12.89;  **untreat-Tg:** 15.59±14.50, 15.8±12.09;  **untreat-WT:** 12.87±11.86, 16.96±16.08 | ANOVA Kruskal-Wallis, Dunn´s multiple comparisons test | **4-OHT-Tg vs 4-OHT-WT:** 0.0011(**), 0.3182;  **4-OHT-Tg vs EtOH-Tg:** 0.0006(***), >0.9999;  **4-OHT-WT vs EtOH-Tg:** >0.9999, 0.7631;  **4-OHT-WT vs untreat-WT:** >0.9999, >0.9999;  **untreat-WT vs untreat-Tg:** >0.9999, >0.9999 |
|  | frequency middle zone (2-11dpt) (n=78-135) | **4-OHT-Tg:**13.31±12.36, 27.44±19.45;  **4-OHT-WT:** 19.41±18, 27.71±16.69;  **EtOH-Tg:** 19.18±14.06, 26.54±15.85;  **untreat-Tg:** 22.90±14.63, 30.47±21.34;  **untreat-WT:** 22.52±15.64, 25.88±17 | ANOVA Kruskal-Wallis, Dunn´s multiple comparisons test | **4-OHT-Tg vs 4-OHT-WT:** 0.0043(**), >0.9999;  **4-OHT-Tg vs EtOH-Tg:** 0.0013(**), >0.9999;  **4-OHT-WT vs EtOH-Tg:** >0.9999, >0.9999;  **4-OHT-WT vs EtOH-Tg:** >0.9999, >0.9999;  **4-OHT-WT vs untreat-WT:** 0.2407, >0.9999;  **untreat-WT vs untreat-Tg:** 0.9999, 0.9016 |
| **5D (swimming 12 well plate)** | total distance moved (2-11dpt) (n=78-135) | **4-OHT-Tg:** 1672±498.5, 1690±885.2;  **4-OHT-WT:** 1402±543.9, 1442±683.5;  **EtOH-Tg:** 1464±535.2, 1648±681.9;  **untreat-Tg:** 1404±531.3, 1553±794.8;  **untreat-WT:** 1451±531, 1511±709.8 | ANOVA Kruskal-Wallis, Dunn´s multiple comparisons test | **4-OHT-Tg vs 4-OHT-WT:** <0.0001 (****), 0.0627;  **4-OHT-Tg vs EtOH-Tg:** 0.0117(*), >0.9999;  **4-OHT-WT vs EtOH-Tg:** 0.7410, 0.1943;  **4-OHT-WT vs untreat-WT:** >0.9999, >0.9999;  **untreat-WT vs untreat-Tg:** >0.9999, >0.9999 |
|  | mean swim speed (2-11dpt) (n=78-135) | **4-OHT-Tg:**4.864±1.457, 4.866±2.5;  **4-OHT-WT:** 4.063±1.585, 4.180±1.974;  **EtOH-Tg:** 4.233±1.54, 4.733±1.933;  **untreat-Tg:** 4.045±1.532, 4.457±2.204;  **untreat-WT:** 4.208±1.517, 4.331±2.035 | ANOVA Kruskal-Wallis, Dunn´s multiple comparisons test | **4-OHT-Tg vs 4-OHT-WT:** <0.0001(****), 0.0562;  **4-OHT-Tg vs EtOH-Tg:** 0.0063(**), >0.9999;  **4-OHT-WT vs EtOH-Tg:** 0.7947, 0.2443;  **4-OHT-WT vs untreat-WT:** >0.9999, >0.9999;  **untreat-WT vs untreat-Tg:** >0.9999, >0.9999 |
|  | max swim speed (2-11dpt) (n=78-135) | **4-OHT-Tg:** 166.8±69.75, 97.56±52,36;  **4-OHT-WT:** 88.61±40.26, 104.6±76.7;  **EtOH-Tg:** 95.34±41.43, 93.45±44.66;  **untreat-Tg:** 79.5±31.62, 84.54±35.53;  **untreat-WT:** 103±63.6, 92.87±46.43 | ANOVA Kruskal-Wallis, Dunn´s multiple comparisons test | **4-OHT-Tg vs 4-OHT-WT:** 0.0004(***), >0.9999;  **4-OHT-Tg vs EtOH-Tg:** 0.0512, >0.9999;  **4-OHT-WT vs EtOH-Tg:** 0.8232, >0.9999;  **4-OHT-WT vs untreat-WT:** 0.6684, >0.9999;  **untreat-WT vs untreat-Tg:** 0.0329(*), >0.9999 |
| **7B (adult number PCs)** | number PCs (2wpt, 4mpt, 1ypt) (n=5) | **Endox:** 213.4±62.76, 663.8±186.2, 785.6±46.82;  **DMSO:** 2578±297.2, 2407±273.4, 2609±286.6 | ordinary one way ANOVA, Šídák's multiple comparisons test | **Endox-2wpt vs Endox-4mpt:**0,0197(*),  **Endox-2wpt vs Endox-1ypt:** 0,0022(**),  **Endox-4mpt vs Endox-1ypt**:0,9467,  **DMSO-2wpt vs DMSO-4mpt:**0,7894,  **DMSO-2wpt vs DMSO-1ypt:**>0,9999,  **DMSO-4mpt vs DMSO-1ypt:**0,6429 |
| **7C (%PCs per areas1-3)** | %PCs/areas 1-3 (2wpt, 4mpt, 1ypt) (n=5) | **Endox 2wpt:** 8.540±5.024, 54.87±3.569, 36.59±7.367; **Endox 4mpt:** 9.192±3.171, 58.18±3.659, 32.63±5.912; **Endox 1ypt:** 11.55±4.172, 57.68±1.990, 30.77±5.839; **DMSO 2wpt:** 29.93±1.747, 41.33±2.810, 28.74±1.760; **DMSO 4mpt:** 28.86±2.324, 37.19±4.180, 33.96±2.445; **DMSO 1ypt:** 28.82±4.374, 35.10±0.7226, 36.08±3.789 |  |  |
| **7D (PC soma size adult)** | diameter PC soma areas 1-3 (2wpt, 4mpt, 1ypt) (n=5) | **Endox 2wpt:** 11.8±2.046, 11.16±2.364, 10.84±2.168;  **Endox 4mpt:** 13.33±2.978, 11.07±2.503, 11.63±2.628; **Endox 1ypt:** 14.63±3.398, 12.03±2.627, 12.23±2.735; **DMSO 2wpt:** 10.47±1.981, 9.069±1.828, 10.11±2.317; **DMSO 4mpt:** 11.71±2.206, 10.08±1.996, 11.08±2.536; **DMSO 1ypt:** 12.01±2.223, 10.65±2.232, 11.35±2.523 | Mann-Whitney test, two tailed | **Endox vs DMSO (2wpt):** <0.0001 (****), <0.0001(****), <0.0001(****);  **Endox vs DMSO (4mpt):** <0.0001 (****), <0.0001(****), <0.0001(****);  **Endox vs DMSO (1ypt):** <0.0001 (****), <0.0001(****), <0.0001(****) |
| **7D (PC soma size adult)** | diameter PC soma areas 1-3 (2wpt, 4mpt, 1ypt) (n=5) | **Endox 2wpt:** 11.8±2.046, 11.16±2.364, 10.84±2.168;  **Endox 4mpt:** 13.33±2.978, 11.07±2.503, 11.63±2.628; **Endox 1ypt:** 14.63±3.398, 12.03±2.627, 12.23±2.735; **DMSO 2wpt:** 10.47±1.981, 9.069±1.828, 10.11±2.317; **DMSO 4mpt:** 11.71±2.206, 10.08±1.996, 11.08±2.536; **DMSO 1ypt:** 12.01±2.223, 10.65±2.232, 11.35±2.523 | Mann-Whitney test, two tailed | **Endox vs DMSO (2wpt):** <0.0001 (****), <0.0001(****), <0.0001(****);  **Endox vs DMSO (4mpt):** <0.0001 (****), <0.0001(****), <0.0001(****);  **Endox vs DMSO (1ypt):** <0.0001 (****), <0.0001(****), <0.0001(****) |
| **7E (% PC type-I adult)** | %PCs soma diameter >10µm areas1-3 (2wpt, 4mpt, 1ypt) (n=5) | **Endox 2wpt**:82.8±8.232, 65.34±7.562, 61.45±8.615;  **Endox 4mpt:** 86.43±7.259, 61.58±13.03, 67.21±11.79; **Endox 1ypt:** 92.27±7.607, 75.15±21.36, 78.21±6.161; **DMSO 2wpt:** 56.68±13.87, 26.89±9.387, 47.04±10.04; **DMSO 4mpt:** 79.35±5.71, 46.79±8.774, 62.16±9.272;  **DMSO 1ypt:** 82.17±1.849, 60.13±8.280, 68.06±5.427 | Unpaired t-test, two tailed | **Endox vs DMSO (2wpt):** 0.068 (**), <0.0001 (****), 0.0441 (*); **Endox vs DMSO (4mpt):** 0.1249, 0.0683, 0.4738;  **Endox vs DMSO (1ypt):** 0.0203 (*), 0.1809, 0.0245 (*) |
| **7G (novel tank test adult)** | % time bottom zone (2wpt-1ypt) (n=15-29) | **Endox:** 84.82±17.79, 87.96±12.47, 81.97±21.83;  **DMSO:** 66.62±20.15, 73.72±17.19, 71.47±26 | Mann-Whitney test, two tailed | **Endox vs DMSO:** 0.0001 (***); 0.0002 (***); 0.1100 |
| **1-fig. suppl. 1A (distance a-p PCL)** | Distance µm a-p PCL (1, 3, 6mpt) | **4-OHT:** 114.875±20.033, 443.298±82.198, 534.836±34.136  **EtOH:** 141.176±42.033, 448.032±40.197, 561.516±62.768 | unpaired T-test two tailed | **4-OHT vs EtOH 1, 3, 6mpt:** 0.2045, 0.9108, 0.4280 |
| **1-fig. suppl. 2D (BrdU+ cells)** | Number BrdU+ cells in the PC layer area | **4-OHT 1-7dpt** (114.2±36.38, 30.2±9.985, 30.67±40.15, 124.3±77.05, 8.33±1.528, 18±2.92),  **EtOH 1-7dpt** (70±45.33, 28.2±15.16, 36.33±18.77, 135.7±55.52, 91.33±8.145, 41±25.16); | unpaired T-test two tailed | **4-OHT vs EtOH 1-7dpt:** 0.0923, 0.8116, 0.8356, 0.8463, <0.0001(****), 0.1962 |
| **1-fig. suppl. 3G (BrdU cells)** | RFP (BrdU) double + cells 26dpt | **4-OHT RFP/BrdU+** (23.13±6.446)  **EtOH RFP/BrdU+** (23.83±3.488) | unpaired T-test two tailed | **4-OHT vs EtOH 26dpt:** 0.8127 |
| **1-fig. suppl. 3H (BrdU/EdU cells)** | RFP (BrdU+/EdU+) double + cells 26dpt | **4-OHT RFP/BrdU+** (3.375±1.302)  **EtOH RFP/BrdU+** (2±0.632) | unpaired T-test two tailed | **4-OHT vs EtOH 26dpt:** 0.0355(*) |
| **2-fig. suppl 1A (ptf1a-anti-blbp cells)** | % ptf1a:GFP-anti-blbp double + | 10.29±2.715 |  |  |
| **2-fig. suppl. 2A (double + PCs)** | Number double + PCs (RFP-GFP) | **4-OHT 0-23dpt** (6±2.898, 5.667±4.131, 7.2±3.493, 3.25±2.217, 13.67±2.805, 13.33±4.885, 19±6-066, 26.33±6.377, 38.67±7.866, 55.2±13.41, 20.8±14.55),  **EtOH 0-23dpt** (30±6.733, 46.2±9.23, 39.57±14.25, 34.33±6.802, 37.8±8.643, 29.2±8.701, 33.33±4.412, 27.4±6.768, 22.5±3.886, 18.63±4.894, 39.8±11.41, 17.83±6.676) | unpaired T-test two tailed | **4-OHT vs EtOH 0-23dpt:** <0.0001(****), <0.0001(****), 0.0002(***), <0.0001(****), 0.0001(***), 0.0024(**), <0.0001(****), 0.0579, 0.2372, <0.0001(****), 0.0862, 0.6638 |
| **2-fig. suppl. 2B (*ptf1a* cells)** | Number *ptf1a+* cells | **4-OHT 0-23dpt:** 191.5±60.75, 232.2±63.62, 240.7±64.11, 234.4±38.63, 163.7±38.03, 252.4±39.73, 212.5±30.91, 213.3±46.12, 207.5±36.84, 207.5±36.84, 489.4±74.03, 458.7±75.14;  **EtOH 0-23dpt:** 287±74.91, 282.6±48.99, 296.8±68.71, 262.2±42.39, 290.8±41.39, 318.2±48.89, 261.2±61.72, 285.8±57.77, 289.2±32.03, 291.7±37.66, 496.6±194.6, 456±34.04 | unpaired T-test two tailed | **4-OHT vs EtOH 0-23dpt:** 0.0565, 0.1820, 0.1949, 0.2897, 0.0089(**), 0.0478(*), 0.1149, 0.0456(*), 0.0022(**), 0.5270, 0.9403, 0.9580 |
| **3-fig. suppl. 1A (reference data)** | Average frequency | **4-21dpf:** 2.83±0.22, 3.24±0.27, 5.21±0.39, 6.87±0.53, 9.18±0.54, 8.05±0.66, 9.01±0.69, 9.79±0.70 |  |  |
| **3-fig. suppl. 1B (reference data)** | Highest frequency | **4-21dpf:** 17.73±1.29, 20.27±2.64, 26.64±2.21, 31.29±2.71, 32.95±2.76, 35.71±3.43, 30.13±2.62, 29±2.57 |  |  |
| **3-fig. suppl. 1C (reference data)** | cc/cs ratio | **4-21dpf**: 0.1263±0.0196, 0.1428±0.0180, 0.0699±0.0102, 0.0439±0.0035, 0.0364±0.0047, 0.0441±0.0051, 0.0324±0.0048, 0.0341±0.0050 |  |  |
| **3-fig. suppl. 1D (PCs)** | Number PCs (0-21dpt) (n=4-10) | **4-OHT:** 65.42±14.85, 35±9.75, 26.25±5.38, 16.89±6.55, 24.5±4.8, 27.8±5.87, 40.33±11.59, 62.5±12.11, 95.14±30.33, 146.375±25.33, 220.29±31.34, 619.6±127.35;  **EtOH:** 283.78±12.35, 300.63±13.24, 322±17.93, 317.5±17.77, 320.4±25.32, 330.375±14.53, 335.88±9.57, 364.22±16.6, 376±40.36, 404±31.83, 463.88±50.86, 906.8±270.6 | 2 way ANOVA Šídák's multiple comparisons test | **0-21dpt 4-OHT vs EtOH:** <0.0001(****), <0.0001(****), <0.0001(****), <0.0001(****), <0.0001(****), <0.0001(****), <0.0001(****), <0.0001(****), <0.0001(****), <0.0001(****), <0.0001(****), <0.0001(****) |
| **3-fig. suppl. 1E (electroph. PCs)** | Average frequency | [see statistics data Fig. 3C] |  | [see data statistics Fig. 3C] |
| **3-fig. suppl. 1F(electroph. PCs)** | Highest frequency | [see statistics data Fig. 3D] |  | [see data statistics Fig. 3D] |
| **3-fig. suppl. 1G (electroph. PCs )** | cc/cs ratio | [see statistics data Fig. 3E] |  | [see data statistics Fig. 3E] |
| **3-fig. suppl. 2A** | PC numbers after repeated ablation (0 dpt – 10 dpst) (n=4-6) | **4-OHT:**113.2±14.582 (n=5), 38.4±2.482 (n=5), 117.2±16.354 (n=5), 261.2±6.651 (n=5), 39.25±15.850 (n=4) 249.67±40.623 (n=6), 763.5±40.876 (n=4)  **EtOH:** 352.8±11.534 (=5), 432.4±11.582 (n=5), 463±11.090 (n=4), 532±24.421 (n=5), 597.2±28.938 (n=5), 761.2±59.254 (n=5), 1192.8±87.014 (=5) | 2- way ANOVA Šídák's multiple comparisons test | **0 dpt – 10 dpst 4-OHT vs EtOH:** <0.0001(****), <0.0001(****), <0.0001(****), <0.0001(****), <0.0001(****), <0.0001(****), <0.0001(****) |
| **3.fig. suppl. 2B/B´** | Average frequency | **4-OHT:** 1.35±0.495 (n=28), 5.24±0.704 (n=32), 8.37±1.069 (n=28), 1.816±0.258 (n=40), 4.744±0.573 (n=25), 8.46±1.076 (n=27)´  **EtOH:** 7.5±1.01 (n=30), 7.927±0.892 (n=30), 8.50±0.797 (n=26), 7.40±0.663 (n=30), 7.27±0.803 (n=25), 8.328±1.242 (n=26) | 2- way ANOVA Šídák's multiple comparisons test | **0 dpt – 10 dpst 4-OHT vs EtOH:** <0.0001 (****), 0.0928, >0.9999, <0.0001 (****), 0.2261, >0.9999 |
| **3-fig. suppl. 2C/C´** | Highest burst frequency | **4-OHT:** 10.11±2.314 (n=28), 16.094±1.547 (n=32), 24.82±1.507 (n=28), 13.3±1.65 (n=40), 21.68±1.894 (n=25), 30.885±2.136 (n=26)  **EtOH:** 31,87±3,088 (n=30), 22,87±1,402 (n=30), 26,28±2,483 (n=25), 25,03±1,759 (n=31), 23,15±1,875 (n=26), 20,23±1,942 (n=26) | 2- way ANOVA Šídák's multiple comparisons test | **0 dpt – 10 dpst 4-OHT vs EtOH:** <0.0001 (****), 0.0799, 0.9971, <0.0001 (****), 0.9972, 0.0025 (**) |
| **3-fig. suppl. 2D/D** | Cs/ss ratio | **4-OHT:** 0,136±0,032 (n=21), 0,142±0,022 (n=32), 0,065±0,012 (n=30), 0,141±0,026 (n=30), 0,160±0,023 (n=24), 0,050±0,010 (n=27)  **EtOH:** 0,065±0,009 (n=30), 0,098±0,013 (n=30), 0,049±0,010 (n=26), 0,067±0,008 (n=31), 0,053±0,009 (n=26), 0,039±0,013 (n=26) | 2- way ANOVA Šídák's multiple comparisons test | **0 dpt – 10 dpst 4-OHT vs EtOH:** 0.0308 (*), 0.2788, 0.9849, 0.0073 (**), 0.0002 (***), 0.9981 |
| **3-fig. suppl. 3B (olig2:GFP cells)** | Olig2:GFP+ cells (15dpt) | **4-OHT:** 157.2±35.42  **EtOH:** 151.5±42.22 | Unpaired t-test two tailed | **4-OHT vs EtOH 15dpt:** 0.8163 |
| **3-fig. suppl. 4A** | PC numbers (1-14 dpt) | **4-OHT:** 21.33±4.46 (n=6), 37±5.053 (n=6), 66.33±7.012 (n=6), 551±16.130 (n=6)  **EtOH:** 452±22.642 (n=4), 496.8±13.735 (n=5), 598±31.087 (n=5), 1151.667±45.693 (n=6) | 2- way ANOVA Šídák's multiple comparisons test | 1-14 dpt 4-OHT vs EtOH: <0.0001(****), <0.0001(****), <0.0001(****), <0.0001(****), |
| **3-fig. suppl. 4B** | Average frequency | [see statistics data Fig. 3H] |  | [see data statistics Fig. 3H] |
| **3-fig. suppl. 4C/D** | Highest burst frequency | **4-OHT:** 29,44±4,551 (n=18), 36,42±5,828 (n=24), 24,61±2,668 (n=28), 34,59±3,671 (n=34), 40,34±5,637 (n=32), 30,41±3,722 (n=34), 25,18±3,330 (n=40)  **EtOH:** 18,31±4,682 (n=15), 21,24±4,061 (n=21), 20,59±3,506 (n=22), 19,09±2,470 (n=23), 27,13±3,856 (n=24), 30,70±4,380 (n=23), 22,38±3,840 (n=24) | 2- way ANOVA Šídák's multiple comparisons test | **0-14 dpt 4-OHT vs EtOH:** 0.6151, 0.1140, 0.9929, 0.0488 (*), 0.1416, >0.9999, 0.9986 |
| **6-fig. suppl. 1B (*in vivo* imaging)** | Intensity RFP fluorescence adult | **before treatment:** 2619±342.7;  **Endox 2wpt-1ypt:** 497±73.22, 1449±249.5, 1849±300.1; **DMSO 2wpt-1ypt:** 2517±389.6, 2338±101.7, 2932±708.7 | Unpaired t-test two tailed | **before treatment vs DMSO 2wpt** (0.5301),  **Endox vs DMSO 2wpt-1ypt** [<0.0001(****), <0.0001(****), 0.0021(**)],  **Endox 2wpt vs 4mpt** [<0.0001(****)],  **Endox 4mpt vs 1ypt** [0.0357(*) |
| **6-fig. suppl. 1C (area PCL)** | Area PC layer adult | **Endox 2wpt-1ypt:** 198462±53841, 347998±58094, 406066±29335;  **DMSO 2wpt-1ypt:** 489777±37715, 711397±95630, 741543±74595 | Mann-Whitney test | **Endox 2wpt vs 4mpt** 0.079(**),  **Endox 4mpt vs 1ypt:** 0.1490,  **Endox vs DMSO:** 2wpt 0.0357(*), 4mpt 0.0079(**) 1ypt 0.0025(**) |
| **6-fig. suppl. 1D (body size)** | Body size adult | **Endox 2wpt-1ypt:** 2.540±0.08944, 2.490±0.2191, 3.614±0.1069;  **DMSO 2wpt-1ypt:** 2.570±0.1204, 2.460±0.2074, 3.600±0.1512 | Mann-Whitney test | **Endox vs DMSO 2wpt-1ypt:** 0.7857, >0.9999, 0.8648 |
| **6-fig. suppl. 1E (NTT)** | Novel tank test adult Mean swim speed | **mean swim speed Endox 2wpt-1ypt:** 4.779±0.6679, 4.407±0.9661, 4.653±1.622;  **mean swim speed DMSO 2wpt-1ypt:** 4.141±1.269, 4.789±0.9121, 4.915±0.9533 | Mann-Whitney test | **mean swim speed Endox vs DMSO 2wpt-1ypt:** 0.0845, 0.3761, 0.6910 |
|  | Novel tank test adult  Total distance moved | **total distance moved Endox 2wpt-1ypt**: 1712±241.6, 1628±344.9, 1680±585.5;  **distance moved DMSO 2wpt-1ypt:** 1589±552.8, 1776±329.7, 1775±343.1 | Unpaired t-test (2wpt, 1ypt)  Mann-Whitney test (4mpt) | **total distance moved Endox vs DMSO 2wpt-1ypt:** 0.5230, 0.3519, 0.6912 |
| **7-fig. suppl. 1B, C (%PCs/areas)** | % PCs / areas CCe adult | **B, C) Endox 2wpt areas 1-3, A-B:** 8.540±5.024, 54.87±3.569, 36.59±7.367, 21.30±8.184, 78.70±8.184;  **Endox 4mpt areas 1-3, A-B:** 9.192±3.171, 58.18±3.659, 32.63±5.912, 24.69±7.278, 75.31±7.278;  **Endox 1ypt areas 1-3, A-B:** 11.55±4.172, 57.68±1.990, 30.77±5.839, 26.18±3.520, 73.82±3.520;  **DMSO 2wpt areas 1-3, A-B:** 29.93±1.747, 41.33±2.810, 28.74±1.760, 51.26±0.9413, 48.74±0.9413;  **DMSO 4mpt areas 1-3, A-B:** 27.96±1.362, 38.98±1.355, 33.06±1.609, 48.56±2.306, 51.44±2.306;  **DMSO 1ypt areas 1-3, A-B:** 29.58±4.654, 34.94±0.7297, 35.48±4.088, 48.24±5.095, 51.76±5.095 | Unpaired t-test two tailed | **B) Endox vs DMSO 2wpt areas 1-3, A-B:** <0.0001(****), 0.0006(***), 0.0619, 0.0002(***), 0.0002(***);  **Endox vs DMSO 4mpt areas 1-3, A-B:** <0.0001(****), 0.6555, 0.0001(***), 0.0001(***);  **Endox vs DMSO 1ypt areas 1-3, A-B:** 0.0005(***), <0.0001(****), 0.2161, 0.0001(***), 0.0001(***) |
|  |  |  | Ordinary one way, ANOVA Šídák's multiple comparisons test | **C) Endox 2wpt:** area1 vs area2 <0.0001(****),  area1 vs area3 <0.0001(****), area2 vs area3 0.0014(**),  area-A vs area-B <0.0001(****);  **Endox 4mpt:** area1 vs area2 <0.0001(****), area1 vs area3 <0.0001(****), area2 vs area3 <0.0001(****), area-A vs area-B <0.0001(****);  **Endox 1ypt:** area1 vs area2 <0.0001(****), area1 vs area3 <0.0001(****), area2 vs area3 <0.0001(****), area-A vs area-B <0.0001(****);  **DMSO 2wpt:** area1 vs area2 <0.0001(****), area1 vs area3 0.7592, area2 vs area3 <0.0001(****), area-A vs area-B 0.1377;  **DMSO 4mpt:** area1 vs area2 0.0004(***), area1 vs area3 0.0306(*), area2 vs area3 0.2696, area-A vs area-B 0.2718;  **DMSO 1ypt:** area1 vs area2 0.1655, area1 vs area3 0.0849, area2 vs area3 0.9954, area-A vs area-B 0.0893 |
| **7-fig. suppl. 1D (PC soma size adult)** | PC soma size adult | **Endox 2wpt areas A-B:** 11.56±2.367, 10.97±2.244;  **Endox 4mpt areas A-B:** 12.45±2.726, 11.14±2.582;  **Endox 1ypt areas A-B:** 13.64±3.232, 11.94±2.595;  **DMSO 2wpt areas A-B:** 10.19±1.995, 9.691±2.210;  **DMSO 4mpt areas A-B:** 11.23±2.085, 10.90±2.493;  **DMSO 1ypt areas A-B:** 11.57±2.291, 10.87±2.410 | Mann-Whitney test | **Endox vs DMSO 2wpt areas A-B:** <0.0001(****), <0.0001(****),  **Endox vs DMSO 4mpt areas A-B:** <0.0001(****), <0.0001(****),  **Endox vs DMSO 1ypt areas A-B:** <0.0001(****), <0.0001(****) |
| **7-fig. suppl. 2B (PC soma size larvae)** | PC soma size larvae (6-23dpt) (n=106-1442) | **4-OHT:** 7.126±1.045, 7.972±1.163, 8.149±1.447, 8.309±1.432  **EtOH:** 6.659±0.8917, 6.926±1.071, 7.727±1.237, 7.452±1.363 | ANOVA Kruskal-Wallis, Dunn´s multiple comparisons test | **4-OHT vs EtOH:** 0.0003 (***), <0.0001 (****), <0.0001 (****), <0.0001 (****) |

**Supplementary Table 1:** Statistical data related to the main figures 1-7 and respective figre supplements. Mean, standard deviation, statistic test applied, ‘n’ number, p value and level of significance are indicated. The reference to the different figures and groups of the comparisons performed in each figure are highlighted in bold. Abbreviations: 4-OHT 4-hydroxytamoxifen, BrdU Bromodeoxyuridine, DMSO dimethyl sulfoxide, dpf days post-fertilization, dpt days post-treatment, EdU Ethynyl-2’-deoxyuridine, Endox Endoxifen, EtOH ethanol, *gfap* glial fibrillary acidic protein, mpf months post-fertilization, mpt months post-treatment, NTT novel tank test, OKR optokinetic response, PC Purkinje cells, PCL Purkinje cell layer, *ptf1a* pancreas associated transcription factor 1a, Tg transgenic, wpt weeks post-treatment, WT wild type, ypf years post-fertilization, ypt years post-treatment.
